# Lectures on Surgical Pathology

**Published:** 1860-01

**Authors:** 


ART. VI.—*Lectures on Surgical Pathology, delivered at the Royal College of Surgeons of England.* By JAMES PAGET, F.R.S., lately Professor of Anatomy and Surgery to the College; Assistant Surgeon and Lecturer on Physiology at St. Bartholomew's Hospital. Second American edition. 8vo., pp. 700. Lindsay & Blakiston: Philadelphia, 1860.

THE work of Mr. Paget is too well known to our readers to require any comments from our hands. It is read and studied wherever the English language is spoken, and there could surely be no better recognition of its claims than this simple circumstance. Since the days of John Hunter, of whom Englishmen and their descendants are so justly proud, no treatise on surgical pathology has appeared that is at all comparable with it, either in point of originality, the accuracy of its doctrines, or the universal favor with which it has been received by the profession. We know some men who swear by Paget, as the Mohammedan does by his Koran; it is his law and gospel; the book of his faith and practice. We were acquainted with a distinguished teacher of surgery, now deceased, who used to tell his students, in the lecture-room, to buy Paget if they were compelled to obtain the money by selling the shirts on their backs. Paget is quoted by everybody—by the hoary-headed professor, and by the medical suckling; and he is evidently destined to have a long and a glorious reign.

Much of this praise, we are satisfied, is deserved; but we are equally convinced that some of it is unmerited. Mr. Paget's work contains some very grave errors; some of his generalizations are hasty, and some of his conclusions quite illogical, if not totally unfounded. We do not deem it necessary to particularize; the present edition contains no new matter, and does not, therefore, call for any special criticism. Mr. Paget had, evidently, nothing to do with its production; it is a mere reprint of the former American edition; and the only thing new about it, that we can discover, is its title-page, with the imprint 1860. The work has now been before the profession for nearly seven years, and it will, no doubt, soon receive a thorough revision from its distinguished author. We shall hail the appearance of a new edition with unmingled gratification. Messrs. Lindsay & Blakiston deserve the thanks of the American reader for having reproduced the lectures in this country.

